# 1-(2-Chloro­eth­yl)-1*H*-pyrazolo­[3,4-*d*]pyrimidin-4(5*H*)-one

**DOI:** 10.1107/S1600536812025184

**Published:** 2012-06-13

**Authors:** Mohammed Iqbal A. Khazi, Nikhath Fathima, Ningaraddi S. Belavagi, Noor Shahina Begum, I. M. Khazi

**Affiliations:** a Department of Chemistry, Karnatak University, Dharwad 580 003, India; b Department of Studies in Chemistry, Bangalore University, Bangalore 560 001, Karnataka, India

## Abstract

In the title compound, C_7_H_7_ClN_4_O, the pyrazolo­pyrimidine ring is essentially planar, the r.m.s. deviation of the fitted atoms being 0.0071 Å. The crystal structure features strong N—H⋯O hydrogen bonds and further consolidated by weak C—H⋯O, C—H⋯N and C—H⋯Cl inter­actions.

## Related literature

For the biological activity of pyrazolo­pyrimidines, see: Carraro *et al.* (2006 ▶). For a related structure, see: Dolzhenko *et al.* (2009 ▶). For the graph-set analysis of hydrogen bonding, see: Bernstein *et al.* (1995 ▶).
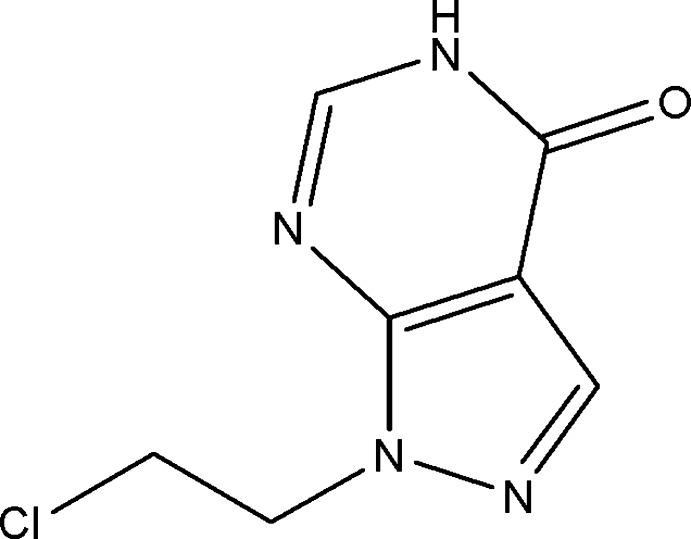


## Experimental

### 

#### Crystal data

C_7_H_7_ClN_4_O
*M* *_r_* = 198.61
Monoclinic, 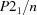

*a* = 4.6448 (1) Å
*b* = 8.0792 (1) Å
*c* = 22.7335 (4) Å
β = 93.554 (1)°
*V* = 851.46 (3) Å^3^
*Z* = 4
Mo *K*α radiation
μ = 0.41 mm^−1^
*T* = 296 K
0.18 × 0.16 × 0.16 mm

#### Data collection

Bruker SMART APEX CCD detector diffractometer
Absorption correction: multi-scan (*SADABS*; Bruker, 1998 ▶) *T*_min_ = 0.930, *T*_max_ = 0.937
7660 measured reflections
1548 independent reflections
1353 reflections with *I* > 2σ(*I*)
*R*_int_ = 0.023

#### Refinement

*R*[*F* ^2^ > 2σ(*F* ^2^)] = 0.035
*wR*(*F* ^2^) = 0.100
*S* = 0.87
1548 reflections
118 parameters
H-atom parameters constrained
Δρ_max_ = 0.19 e Å^−3^
Δρ_min_ = −0.37 e Å^−3^

### 

Data collection: *SMART* (Bruker, 1998 ▶); cell refinement: *SAINT-Plus* (Bruker, 1998 ▶); data reduction: *SAINT-Plus*; program(s) used to solve structure: *SHELXS97* (Sheldrick, 2008 ▶); program(s) used to refine structure: *SHELXL97* (Sheldrick, 2008 ▶); molecular graphics: *ORTEP-3* (Farrugia, 1997 ▶) and *CAMERON* (Watkin *et al.*, 1996 ▶); software used to prepare material for publication: *WinGX* (Farrugia, 1999 ▶).

## Supplementary Material

Crystal structure: contains datablock(s) global, I. DOI: 10.1107/S1600536812025184/pv2552sup1.cif

Structure factors: contains datablock(s) I. DOI: 10.1107/S1600536812025184/pv2552Isup2.hkl

Supplementary material file. DOI: 10.1107/S1600536812025184/pv2552Isup3.cml

Additional supplementary materials:  crystallographic information; 3D view; checkCIF report

## Figures and Tables

**Table 1:** Hydrogen-bond geometry (Å, °)

*D*—H⋯*A*	*D*—H	H⋯*A*	*D*⋯*A*	*D*—H⋯*A*
N1—H1⋯O1^i^	0.86	1.96	2.810 (2)	170
C5—H5*A*⋯N4^ii^	0.93	2.79	3.676 (2)	160
C2—H2*A*⋯Cl1^iii^	0.97	2.84	3.779 (2)	164
C2—H2*B*⋯N2^iv^	0.97	2.59	3.463 (2)	150
C3—H3⋯O1^v^	0.93	2.35	3.254 (2)	163

Symmetry codes: (i) 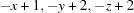; (ii) 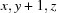; (iii) 
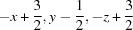
; (iv) 
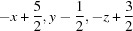
; (v) 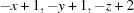.

**Fig. 1.**
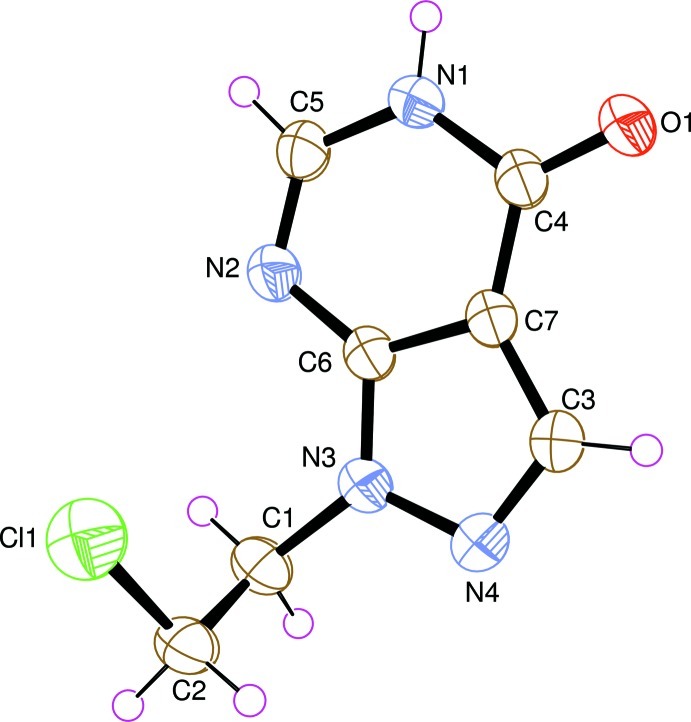
The molecular structure of the title compound with the atom numbering scheme. Displacement ellipsoids are drawn at the 50% probability level. H atoms are presented as small spheres of arbitrary radius.

**Fig. 2.**
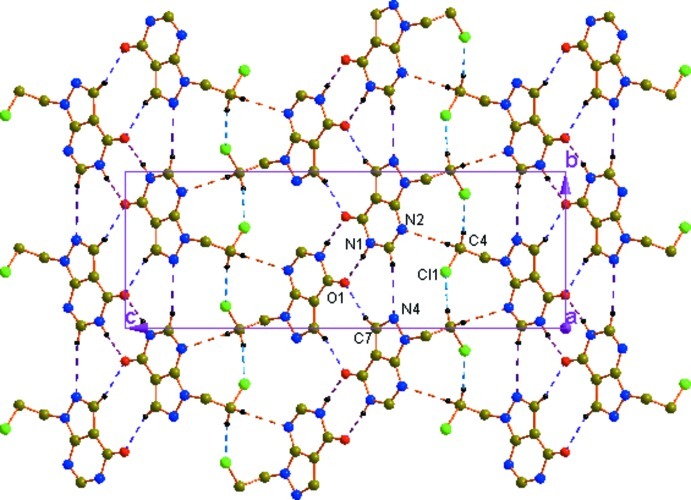
A view of the intermolecular hydrogen bonds (dotted lines) in the crystal structure of the title compound. H atoms non participating in H-bonding were ommitted for clarity.

## supplementary crystallographic information

### Comment

Pyrazolo[3,4-*d*]pyrimidines are purine analogues which exhibit a number of pharmacological properties such as antitryproliferative (Carraro *et al.*, 2006).

In the title compound (Fig. 1), the fused pyrazolopyrimidine ring is substituted with 2-chloro-ethyl group on one side and the oxo group on the other side. The pyrazolopyrimidine ring is planar with the maximum deviation from the mean statistical plane being 0.0115 (14) Å for C3. The *cis* orientation of 2-chloro-ethyl group with respect to the C2—N2 bond is described by the torsion angle N2—C2—N3—C3, -2.204 (4)°.

The crystal structure is stabilized by some interesting features that comprise of intermolecular N—H···O, C—H···O, C—H···N and C—H···Cl interactions (Fig. 2 and Tab. 1). The C—H···O and the N—H···O interactions result in centrosymmetric head-to-head dimers corresponding to the graph set *R*^2^_2_(10) and *R*^2^_2_(8) motif (Bernstein *et al.*, 1995). There are two types of C—H···N interactions, one of which forms a helix, the other forms sheets along the crystallographic *b*-axis. The C—H···Cl intermolecular interaction result in one dimensional molecular chain along *b*-axis. The bond lengths and bond angles in the title molecule agree very well with the corresponding bond distances and bond angles reported in a closely related compound (Dolzhenko *et al.*, 2009).

### Experimental

A mixture of 5-amino-1-(2-chloro-ethyl)-1*H*-pyrazole-4-carbonitrile (1 g, 5.8 mmol) and formic acid (15 ml) was heated under reflux for 10 h. The excess of formic acid was removed under reduced pressure and the solid separated was washed with water and recrystallized from ethanol. (Yield = 0.86 g, 75% and m.p. = 470–472 K).

### Refinement

The H atoms were placed at calculated positions in the riding model approximation with N—H = 0.86 Å and C—H = 0.93, and 0.97 Å for aryl and methylene H-atoms respectively, with *U*_iso_(H) = 1.2*U*_eq_(N/C).

### Crystal data

**Table:**

C_7_H_7_ClN_4_O	*F*(000) = 416
*M**_r_* = 198.61	*D*_x_ = 1.565 Mg m^−^^3^
Monoclinic, *P*2_1_/*n*	Mo *K*α radiation, λ = 0.71073 Å
Hall symbol: -P 2yn	Cell parameters from 1548 reflections
*a* = 4.6448 (1) Å	θ = 1.8–25.2°
*b* = 8.0792 (1) Å	µ = 0.41 mm^−^^1^
*c* = 22.7335 (4) Å	*T* = 296 K
β = 93.554 (1)°	Block, yellow
*V* = 851.46 (3) Å^3^	0.18 × 0.16 × 0.16 mm
*Z* = 4	

### Data collection

**Table:**

Bruker SMART APEX CCD detector diffractometer	1548 independent reflections
Radiation source: fine-focus sealed tube	1353 reflections with *I* > 2σ(*I*)
Graphite monochromator	*R*_int_ = 0.023
ω scans	θ_max_ = 25.2°, θ_min_ = 1.8°
Absorption correction: multi-scan (*SADABS*; Bruker, 1998)	*h* = −5→5
*T*_min_ = 0.930, *T*_max_ = 0.937	*k* = −9→9
7660 measured reflections	*l* = −27→25

### Refinement

**Table:**

Refinement on *F*^2^	Primary atom site location: structure-invariant direct methods
Least-squares matrix: full	Secondary atom site location: difference Fourier map
*R*[*F*^2^ > 2σ(*F*^2^)] = 0.035	Hydrogen site location: inferred from neighbouring sites
*wR*(*F*^2^) = 0.100	H-atom parameters constrained
*S* = 0.87	*w* = 1/[σ^2^(*F*_o_^2^) + (0.0642*P*)^2^ + 0.5291*P*] where *P* = (*F*_o_^2^ + 2*F*_c_^2^)/3
1548 reflections	(Δ/σ)_max_ = 0.001
118 parameters	Δρ_max_ = 0.19 e Å^−^^3^
0 restraints	Δρ_min_ = −0.37 e Å^−^^3^

### Special details

**Table:**

Geometry. All s.u.'s (except the s.u. in the dihedral angle between two l.s. planes) are estimated using the full covariance matrix. The cell s.u.'s are taken into account individually in the estimation of s.u.'s in distances, angles and torsion angles; correlations between s.u.'s in cell parameters are only used when they are defined by crystal symmetry. An approximate (isotropic) treatment of cell s.u.'s is used for estimating s.u.'s involving l.s. planes.
Refinement. Refinement of *F*^2^ against ALL reflections. The weighted *R*-factor *wR* and goodness of fit *S* are based on *F*^2^, conventional *R*-factors *R* are based on *F*, with *F* set to zero for negative *F*^2^. The threshold expression of *F*^2^ > 2σ(*F*^2^) is used only for calculating *R*-factors(gt) *etc*. and is not relevant to the choice of reflections for refinement. *R*-factors based on *F*^2^ are statistically about twice as large as those based on *F*, and *R*- factors based on ALL data will be even larger.

### Fractional atomic coordinates and isotropic or equivalent isotropic displacement parameters (Å^2^)

**Table:**

	*x*	*y*	*z*	*U*_iso_*/*U*_eq_	
Cl1	0.89765 (12)	0.64259 (8)	0.72864 (2)	0.0600 (2)	
O1	0.5103 (3)	0.78930 (17)	0.99906 (6)	0.0516 (4)	
N1	0.7733 (3)	0.95763 (18)	0.94256 (6)	0.0381 (4)	
H1	0.7021	1.0429	0.9590	0.046*	
N2	1.0887 (3)	0.86963 (19)	0.87124 (7)	0.0396 (4)	
N3	1.0967 (3)	0.57267 (18)	0.86397 (6)	0.0376 (4)	
N4	0.9763 (3)	0.43809 (19)	0.88974 (7)	0.0434 (4)	
C5	0.9640 (4)	0.9842 (2)	0.90068 (8)	0.0401 (4)	
H5A	1.0092	1.0935	0.8924	0.048*	
C6	1.0057 (4)	0.7151 (2)	0.88711 (7)	0.0331 (4)	
C1	1.2904 (4)	0.5504 (3)	0.81692 (8)	0.0418 (4)	
H1A	1.4385	0.4711	0.8295	0.050*	
H1B	1.3848	0.6548	0.8096	0.050*	
C2	1.1398 (4)	0.4907 (2)	0.76054 (8)	0.0442 (5)	
H2A	1.0330	0.3907	0.7683	0.053*	
H2B	1.2826	0.4635	0.7327	0.053*	
C4	0.6848 (4)	0.8026 (2)	0.96076 (7)	0.0371 (4)	
C7	0.8174 (4)	0.6729 (2)	0.92974 (7)	0.0349 (4)	
C3	0.8092 (4)	0.4993 (2)	0.92932 (8)	0.0424 (4)	
H3	0.7002	0.4356	0.9538	0.051*	

### Atomic displacement parameters (Å^2^)

**Table:**

	*U*^11^	*U*^22^	*U*^33^	*U*^12^	*U*^13^	*U*^23^
Cl1	0.0545 (3)	0.0640 (4)	0.0607 (4)	0.0019 (2)	−0.0027 (3)	0.0011 (3)
O1	0.0692 (9)	0.0403 (8)	0.0488 (8)	−0.0011 (6)	0.0335 (7)	−0.0006 (6)
N1	0.0486 (8)	0.0314 (8)	0.0355 (8)	0.0000 (6)	0.0122 (6)	−0.0036 (6)
N2	0.0475 (9)	0.0357 (8)	0.0370 (8)	−0.0044 (7)	0.0137 (7)	−0.0006 (6)
N3	0.0437 (8)	0.0351 (8)	0.0354 (8)	0.0002 (6)	0.0121 (6)	−0.0014 (6)
N4	0.0550 (9)	0.0326 (8)	0.0436 (9)	0.0000 (7)	0.0117 (7)	0.0026 (7)
C5	0.0489 (10)	0.0352 (10)	0.0372 (9)	−0.0061 (8)	0.0098 (8)	0.0021 (8)
C6	0.0360 (9)	0.0343 (9)	0.0295 (8)	−0.0008 (7)	0.0060 (7)	−0.0005 (7)
C1	0.0397 (9)	0.0451 (11)	0.0418 (10)	0.0050 (8)	0.0132 (8)	−0.0037 (8)
C2	0.0505 (11)	0.0395 (11)	0.0442 (10)	0.0004 (8)	0.0159 (8)	−0.0060 (8)
C4	0.0452 (10)	0.0365 (10)	0.0304 (9)	−0.0030 (8)	0.0088 (7)	−0.0002 (7)
C7	0.0413 (9)	0.0347 (9)	0.0293 (8)	−0.0019 (7)	0.0076 (7)	0.0006 (7)
C3	0.0528 (11)	0.0364 (10)	0.0393 (10)	−0.0025 (8)	0.0141 (8)	0.0033 (8)

### Geometric parameters (Å, º)

**Table:**

Cl1—C2	1.788 (2)	C5—H5A	0.9300
O1—C4	1.231 (2)	C6—C7	1.388 (2)
N1—C5	1.357 (2)	C1—C2	1.501 (3)
N1—C4	1.389 (2)	C1—H1A	0.9700
N1—H1	0.8600	C1—H1B	0.9700
N2—C5	1.299 (2)	C2—H2A	0.9700
N2—C6	1.362 (2)	C2—H2B	0.9700
N3—C6	1.344 (2)	C4—C7	1.424 (2)
N3—N4	1.371 (2)	C7—C3	1.403 (3)
N3—C1	1.451 (2)	C3—H3	0.9300
N4—C3	1.320 (2)		
			
C5—N1—C4	124.71 (15)	C2—C1—H1B	109.0
C5—N1—H1	117.6	H1A—C1—H1B	107.8
C4—N1—H1	117.6	C1—C2—Cl1	112.00 (14)
C5—N2—C6	111.97 (15)	C1—C2—H2A	109.2
C6—N3—N4	111.42 (13)	Cl1—C2—H2A	109.2
C6—N3—C1	128.26 (15)	C1—C2—H2B	109.2
N4—N3—C1	120.30 (15)	Cl1—C2—H2B	109.2
C3—N4—N3	105.48 (15)	H2A—C2—H2B	107.9
N2—C5—N1	125.45 (17)	O1—C4—N1	120.63 (16)
N2—C5—H5A	117.3	O1—C4—C7	127.58 (17)
N1—C5—H5A	117.3	N1—C4—C7	111.79 (14)
N3—C6—N2	125.45 (15)	C6—C7—C3	105.00 (16)
N3—C6—C7	106.86 (15)	C6—C7—C4	118.38 (16)
N2—C6—C7	127.68 (16)	C3—C7—C4	136.61 (17)
N3—C1—C2	113.08 (15)	N4—C3—C7	111.23 (16)
N3—C1—H1A	109.0	N4—C3—H3	124.4
C2—C1—H1A	109.0	C7—C3—H3	124.4
N3—C1—H1B	109.0		
			
C6—N3—N4—C3	−0.1 (2)	C5—N1—C4—O1	−179.89 (18)
C1—N3—N4—C3	−178.72 (16)	C5—N1—C4—C7	−0.4 (2)
C6—N2—C5—N1	0.1 (3)	N3—C6—C7—C3	0.2 (2)
C4—N1—C5—N2	0.7 (3)	N2—C6—C7—C3	−179.13 (18)
N4—N3—C6—N2	179.28 (17)	N3—C6—C7—C4	−179.02 (15)
C1—N3—C6—N2	−2.2 (3)	N2—C6—C7—C4	1.6 (3)
N4—N3—C6—C7	−0.1 (2)	O1—C4—C7—C6	178.81 (19)
C1—N3—C6—C7	178.40 (17)	N1—C4—C7—C6	−0.6 (2)
C5—N2—C6—N3	179.47 (17)	O1—C4—C7—C3	−0.1 (4)
C5—N2—C6—C7	−1.3 (3)	N1—C4—C7—C3	−179.6 (2)
C6—N3—C1—C2	−108.1 (2)	N3—N4—C3—C7	0.2 (2)
N4—N3—C1—C2	70.3 (2)	C6—C7—C3—N4	−0.3 (2)
N3—C1—C2—Cl1	66.78 (19)	C4—C7—C3—N4	178.7 (2)

### Hydrogen-bond geometry (Å, º)

**Table:**

*D*—H···*A*	*D*—H	H···*A*	*D*···*A*	*D*—H···*A*
N1—H1···O1^i^	0.86	1.96	2.810 (2)	170
C5—H5*A*···N4^ii^	0.93	2.79	3.676 (2)	160
C2—H2*A*···Cl1^iii^	0.97	2.84	3.779 (2)	164
C2—H2*B*···N2^iv^	0.97	2.59	3.463 (2)	150
C3—H3···O1^v^	0.93	2.35	3.254 (2)	163

Symmetry codes: (i) −*x*+1, −*y*+2, −*z*+2; (ii) *x*, *y*+1, *z*; (iii) −*x*+3/2, *y*−1/2, −*z*+3/2; (iv) −*x*+5/2, *y*−1/2, −*z*+3/2; (v) −*x*+1, −*y*+1, −*z*+2.
